# Hereditary Angioedema Type II: First Presentation in Adulthood with Recurrent Severe Abdominal Pain

**DOI:** 10.1155/2018/7435870

**Published:** 2018-10-29

**Authors:** Mohamed Abuzakouk, Nada AlMahmeed, Esat Memisoglu, Martine McManus, Aydamir Alrakawi

**Affiliations:** Cleveland Clinic Abu Dhabi, UAE

## Abstract

A 27-year-old Emirate man presented to Cleveland Clinic Abu Dhabi emergency department with a 4 year history of recurrent episodes of severe swellings affecting different parts of his body. He used to get 2 swelling episodes every week affecting either his face, hands, feet or scrotum and severe abdominal pain twice a week. Abdominal CT scan and a colonoscopy showed bowel wall oedema. There was no family history of similar complaint or of hereditary angioedema (HAE). Complement studies confirmed the diagnosis of HAE type II. He was commenced on danazol 100 mg twice daily and his symptoms resolved. This case report highlights the importance of considering HAE in patients with recurrent unexplained abdominal pain even in the absence of positive family history of HAE.

## 1. Introduction

Hereditary angioedema (HAE) is a rare, potentially fatal genetic disease with an estimated prevalence of 1/10 000–1/100 000 inhabitants [1]. The disorder is due to mutations in one of the two alleles of C1-INH gene, SERPING1 resulting in reduced plasma levels of C1-INH and instability of the contact system with facilitated release of bradykinin, a key mediator of angioedema [1]. The pathogenesis of the two most common types of this condition is the lack of or abnormal function of C1 esterase inhibitor (C1-INH) [1, 2]. Type I affects approximately 85% of cases and patients have low serum levels of C1-INH. Type II constitutes about 15% of cases and patients have normal or elevated levels of nonfunctional C1-INH. Type III HAE with normal C1-INH is a variant where all measurements of C1-INH are normal but attacks of angioedema are similar to those in types I and II. The mechanism of this condition is not fully understood [1, 2].

The clinical manifestations of HAE include recurrent attacks of non-pitting swelling of the face, lips, tongue, throat, extremities and genitals. In addition to the cutaneous and soft tissue involvement, more than 80% of patients present with gastrointestinal symptoms. This can often be misdiagnosed for ischemic colitis, infectious aetiologies and inflammatory bowel disease. The difficulty in recognizing gastrointestinal symptoms as being related to HAE often leads to a delay in diagnosis and to unnecessary investigations and surgical procedures.

## 2. Case Presentation

A 27-year-old Emirati male presented to Cleveland Clinic Abu Dhabi (CCAD) emergency department (ED) for the first time in Sept 2015 complaining of severe abdominal pain. The pain has been episodic for the last 4 years and had significantly affected his work and family life. He was seen and admitted to multiple hospitals across Abu Dhabi, including our own, attended different specialists, and underwent a wide range of investigations including blood tests (CBC and differential count, liver and renal profiles and CRP), gastroscopies, colonoscopies, and CT scans and a laparoscopy. The results of all his clinical assessments and investigations did not show any sign.

On one of his acute admissions to CCAD, the immunologist was asked to review the patient. Detailed examination of the patient's medical history starting from the onset of symptoms reveled that he used to get 2 swelling episodes every week affecting his face, hands, feet or scrotum and severe abdominal pain twice a week. These swelling episodes and abdominal pain appeared suddenly without any obvious triggering factor, developed over 36 hours and resolved spontaneously in 5-7 days without any medication including analgesics. He reported no laryngeal swellings or respiratory compromise. He denied any fevers, night sweats, weight loss, change in bowel habits or blood in his stools. His swelling episodes were occasionally associated with non-pruritic red skin rash that was mistaken for chronic urticaria for which he was treated with Omalizumab (300mg every 4 weeks) for 9 months without any benefit. Moreover, his response to different types of analgesia, high dose antihistamines, antibiotics and corticosteroids was unsatisfactory. He is a thalassemia carrier; otherwise he is fit and healthy and has no past medical history of note. He has no family history of immunodeficiency, inflammatory bowel disease, autoimmunity or FMF. On examination, he was in pain and his abdomen was soft, tender with guarding and decreased bowel sounds. There was no rebound, rigidity, distension or ascites. He had no peripheral swellings.

**Figure 1:**
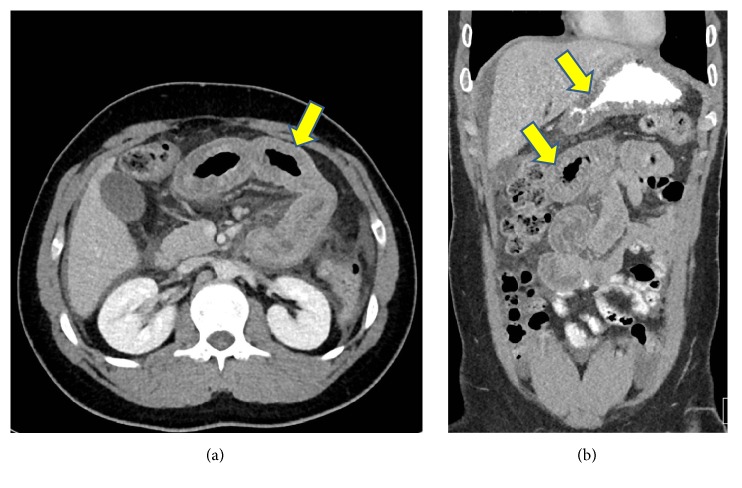
Abdominal CT scan during an acute attack showing long segments of enhancing mucosal thickening involving the proximal jejunum (arrows in a and b) and gastric mucosa (upper arrow in b).

**Figure 2:**
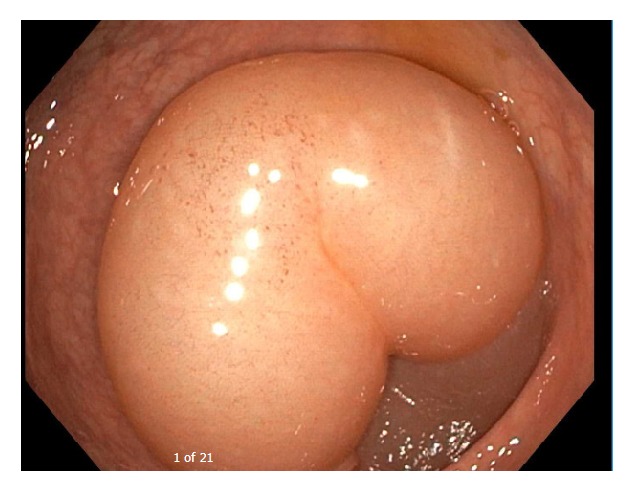
Colonoscopy during an acute attack showing severe mucosal edema in the transverse colon with occlusion of the lumen.

During his acute admission to CCAD, a review of his blood tests was performed that showed reduced C4 and absent C1 inhibitor function (Table). These tests were performed a month prior to his admission but were not followed up. His abdominal CT scan showed diffuse swelling and long segment of enhancing mucosal thickening involving the proximal jejunum and gastric mucosa with minimal free abdominal fluid (Figure 1). In addition, he had a colonoscopy which showed severe mucosal edema in the transverse colon with occlusion of the lumen (Figure 2). A provisional diagnosis of HAE was made based on his limited complement studies. As he had severe abdominal pain for 24 hours prior to his hospital admission, C1 inhibitor concentrate (2000 units IV over 10 minutes) was administered and within 2 hours his pain had almost resolved.

After his recovery, he underwent detailed immunological investigations that revealed markedly reduced C4 level and absent C1 inhibitor function (performed manually, read on Shimadzu UV-1700 equipment) with normal C3 and C1q levels and raised C1 inhibitor serum levels (Table 1). His ENA, total immunoglobulin, CBC and differential count, serum protein electrophoresis, liver function tests, hepatitis serology, lipase, amylase, tissue transglutaminase, stools tests and urinalysis did not show any significant abnormality.

He was diagnosed with type 2 HAE based on his abnormal complement studies (Table 1) and was commenced on tranexamic acid for 3 months. He did not want to start with attenuated androgens because he was concerned about their adverse effects. However, he continued to get abdominal pain even when the dose of tranexamic acid was increased to 3 grams daily. He was then switched to danazol 100 mg twice daily with complete resolution of his abdominal symptoms.

## 3. Discussion

Hereditary angioedema is a disease characterized by recurrent episodes of angioedema; most often affect the skin and mucosal tissues of the upper respiratory and gastrointestinal tracts. During episodes of angioedema in patients with HAE, plasma bradykinin levels have been shown to be seven fold higher than normal [2]. In bradykinin-mediated angioedema, histamine and other mast cell mediators are not directly involved which explains the lack of response to antihistamines and distinguishes this form of angioedema from the histamine-mediated angioedema that is seen in allergic reactions A wide range of associated and prodromal symptoms have been reported in patients with HAE in recent studies [3, 4]. Among these, the most specific symptom is erythema marginatum (EM), a reticular and serpiginous, usually non-pruritic skin rash. EM is distinct from urticaria, which is pruritic, red, raised and transient rash with widespread wheals that is absent in HAE. It is highly likely that our patient had EM that was mistaken for chronic urticaria and that explains his lack of response to antihistamines and Omalizumab.

Gastrointestinal tract involvement is an important feature, often the most common symptom missed in HAE. Crampy and colicky abdominal pain is present in 43-93% of cases [5]. The pain can present acutely or as constantly recurring pain for many years without any associated respiratory or cutaneous involvement. In addition, 88% of patients with gastrointestinal swelling usually experience nausea and vomiting [6], 65 % have diarrhea [7] and 72% have abdominal distension [8]. Constipation can occur if the swelling is located in the lower gut or the colon. Other gastrointestinal symptoms including pancreatitis and intussusception are rare [9]. The difficulty in recognizing gastrointestinal symptoms as being related to HAE often leads to a delay in diagnosis and to unnecessary investigations, surgical procedures and poor quality of life. In our patient, there was a delay of diagnosis of about 4 years which had a significant impact on his work and family life. Therefore, HAE should be suspected in a patient with unexplained recurrent episodes of self-limited, colicky and crampy abdominal pain, ascites or diarrhoea.

In HAE, obtaining a detailed medical and family history and performing a thorough physical examination is crucial to help direct the appropriate diagnostic testing. There are reports of adult patient with type I HAE presenting with recurrent abdominal pain with positive family history of HAE [9–11]. However, cases of angioedema with no family history but with functionally low levels of C1 inhibitor and recurrent attacks are often observed. De novo C1inhibitor mutations and exon deletions account for at least 25% of all unrelated cases of angioedema [12]. Our patient with the rare form of the disorder had no family history of HAE making the diagnosis even more difficult to establish. To confirm the diagnosis of HAE, the recommended initial screening laboratory tests include serum C4, C1-INH levels, C1-INH function and serumC1q level [1, 9]. The C4 level is typically low in most cases of HAE and is the quickest and most readily available screening test. Measurement of C4 antigen can exclude diagnosis of C1-INH-HAE with an accuracy of > 95%. Therefore, this parameter should be considered for initial screening in differential diagnosis of angioedema [13]. The findings of low C4 and low C1-INH levels and activity and a normal C1q level are confirmatory tests for HAE type 1, while type 2 HAE laboratory findings would reveal a normal or raised C1-INH and C1q levels with low C1-INH functional activity. Complement studies should be repeated after one month to confirm the results and diagnosis. Our patient had abnormal complement studies on several occasions. Genetic testing for HAE is relatively complicated because of the heterogeneity of mutations responsible for C1-INH deficiency and is not required in the majority of patients. It is helpful during the first year of age, when C1-INH plasma levels may be falsely low and to distinguish C1-INH-AAE when diagnosis is not clear-cut [14].

The management of HAE includes patient education and support, managing acute attacks, short-term prophylaxis for procedures that place the patient at risk of an acute attack, and long-term management for those patients whose attack rate is high or impacts adversely on ability to work and conduct daily life [1]. Acute attacks should be treated with C1-INH concentrate, ecallantide (Kallikrein inhibitor), or icatibant (Bradykinin-receptor antagonist) [15]. If a C1-INH concentrate, ecallantide, or icatibant is not available, attacks should be treated with solvent detergent-treated plasma (SDP). If SDP is not available, then attacks should be treated with fresh frozen plasma. The use of antifibrinolytics (e.g., tranexamic acid) or androgens (e.g. danazol) is not recommended for acute treatment of HAE attacks, as these drugs have no or only minimal effects [15].

The mainstay of treatment of hereditary angioedema is long term prophylaxis to prevent acute attacks. Medications used include regular injections of C1-INH concentrate, attenuated androgens (danazol, stanozolol) or antifibrinolytics agents (tranexamic acid, epsilon aminocaproic acid). Our patient did not respond to tranexamic acid but his symptoms were controlled on danazol. Antifibrinolytics agents are less predictably effective for preventing HAE episodes compared with C1-INH concentrate or androgens. However, these agents can be useful for long-term prophylaxis in growing children and possibly in women who are pregnant or planning to become pregnant in the near future, in whom androgens should not be used [15]. Transient prophylaxis is required prior to surgical procedures particularly dental procedures in patients with HAE, as oedema involving the upper airways may lead to asphyxia. This is best achieved by administering C1-INH concentrate 24 hours before the procedure [15].

Despite standard of care, HAE patients still have frequent and painful attacks causing significant physical and emotional impairment both during and between attacks. Therefore, these patients will require continuous support and understanding to cope better with their disease which may eventually help in the clinical management of their life-long condition [16]. Initially, our patient was in denial and refused to accept his diagnosis and kept missing his medications. However, with continuous medical support and education he came to terms with his condition and accepted that he requires long-term treatment. He has not had any acute admission to hospital because of severe abdominal pain since commencing danazol and he currently leads a normal happy life.

## 4. Conclusion

Hereditary angioedema is a disease that is often misdiagnosed. Delayed diagnosis leads to unnecessary investigations and procedures with significant social and psychological burden, especially in those presenting with abdominal symptoms. HAE should be suspected in all patients presenting with unexplained episodic abdominal pain even in the absence of positive family history of HAE. Measuring C-1 INH level and function and C4 levels are the mainstay diagnostic tests. HAE is a treatable condition and with proper follow up and support, patients are able to lead a normal life.

## Figures and Tables

**Table 1:** Complement studies.

	Ref. Range	12/08/2016	15/09/2016	3/11/2016	29/04/ 2018
C1 Esterase Inhibitor function	41->67%	2	29	35	29
C1 Esterase Inhibitor serum level	210 - 390 mg/L	Not done	450	610	780
C4	9 - 36 mg/dL	2	3	5	6
C3	90 - 180 mg/dL	114	104	140	134
Serum C1q	303 - 610 nmol/L	-	-	377	-
